# Synthesis of Environmentally Friendly Acrylonitrile Butadiene Styrene Resin with Low VOC

**DOI:** 10.3390/ma13071663

**Published:** 2020-04-03

**Authors:** Licheng Fan, Lijuan Wei, Yongfei Zhu, Yibo Wang, Jianmin Fei, Yang Li

**Affiliations:** 1 North Huajin Chemical Industries Group, Panjin 124021, China; fanlicheng777@163.com (L.F.); weilijuan0605@163.com (L.W.); zhuyongfei701@163.com (Y.Z.); jszxwyb@163.com (Y.W.); jthfeijianmin@163.com (J.F.); 2 Department of Polymer Science and Engineering, Dalian University of Technology, Dalian 116024, China

**Keywords:** ABS resin with low VOC, chain transfer agents, extracting agent, orthogonal design

## Abstract

Most acrylonitrile butadiene styrene (ABS) resin is plagued by an unpleasant odor attributed to the high residual volatile organic compound (VOC) content. This paper primarily aimed to solve the odor issue of ABS resin by effectively reducing the VOC content. To that end, a synthesis of ABS resins was optimized through a supercritical extraction process while evaluating multiple novel chain transfer agents (linear dimer of α-methyl-styrene, methyl 3-mercaptopropionate, and dodecyl mercaptan). ABS resin obtained through a α-methyl-styrene chain transfer agent demonstrated the lowest odor. Moreover, it had the least amount of VOC content which was three times lower than when dodecyl mercaptan was employed. To improve the supercritical extraction process, an orthogonal test was designed to optimize four main process parameters: extrusion temperature, residence time, vacuum degree and extractant dosage. The most optimal conditions were found to be 250 °C extrusion temperature, one minute residence time, vacuum degree of minus 99 KPa, and 1.5% CO_2_ extractant dosage.

## 1. Introduction

Acrylonitrile butadiene styrene (ABS) resin has recently attracted significant attention from the consumer markets, especially automobiles, small household appliances, and 3D printing. Consumers are becoming more concerned about their health and prefer to use items fabricated from materials without an odor or harmful volatile organic compounds (VOCs). Additionally, an increasing number of stringent environmental and consumer-related regulations are now introduced worldwide on the control of odor and VOC content in ABS products. Thus, the development of ABS resin with low odor and low VOC content has become a major research area in the plastics industry [1,2].

Several research efforts on how to reduce VOC as well as the odor of the resin and their blends were reported in the literature. Villberg et al. [3] reported that the source of polypropylene (PP) odor was volatile carbonyl compounds (such as aldehydes, ketones, and esters). According to Reingruber et al. [4], such emissions from PP products during their production might be very harmful, which, in turn, greatly limits PPs application in automotive interiors. Wrona et al. [5] quantitatively analyzed odor components released by the oxidation and biodegradation of polyethylene (PE). Hodgson et al. [6] applied gas chromatography, olfactory sensing and electronic arm techniques to determine the sources of VOCs during PE production. Carbonyl compounds were determined to be the main causes of odor in polyolefins [7]. The majority of these carbonyl compounds are derived from oxidation reactions and were present in the raw material even before PP processing and modification. However, research regarding ABS resins with low VOC content is still lacking. Additionally, only a few companies in the world (such as Dow Chemical) are capable of producing ABS resin of low VOC and odor.

It is noted that supercritical fluids have demonstrated special properties to remove residual and volatile compounds from the polymers [8]. Supercritical fluids were first applied as scavengers during devolatilization (which includes extruding, flash and stripping devolatilization techniques) in the 1990s. Miyakawa et al. [9] applied supercritical CO_2_ to remove volatiles from polystyrene (PS). Krupinski and McQueen [10] injected the supercritical CO_2_ into the molten state of PS through the injection hole at the end of the extruder, after which the residual monomer was removed by a flash tank. Odell [11] synthesized polycarbonate (PC) at high CO_2_ pressure by applying supercritical CO_2_ for residual monomer and by-product extraction.

Though supercritical extraction has been researched for the improvement of many resin products, very few studies have been applied for ABS plastics to remove residuals and volatiles. Therefore, the main goal of this paper was to demonstrate the synthesis of low VOC/odor ABS resin through the supercritical extraction process. We first applied multiple chain transfer agents in the polymerization phase. Then supercritical extraction technology was adopted as a post-polymerization process to remove small molecules and to reduce VOC content in the final product.

## 2. Materials and Methods

### 2.1. Synthesis

**Figure 1:**
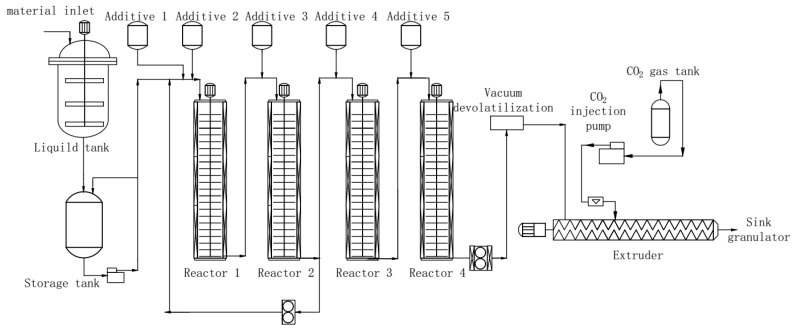
Schematics of preparation of acrylonitrile butadiene styrene (ABS) resin with low volatile organic compound (VOC) using the supercritical extraction method.

Chopped rubber, ethylbenzene, acrylonitrile and styrene were placed into a sol tank maintained at a consistent temperature of 25 °C and mixed together until uniform glue liquid was obtained. For a continuous bulk polymerization, it was placed into one section of a polymerization reactor, while the initiator, chain transfer agent, and silicone were placed in the other three sections (see Figure 1). As-obtained polymerized resin was then transferred to a supercritical extraction device for devolatilization and pelletization. Ethylbenzene was used as the solvent. The resulting material was ABS resin pellets with low VOC as well as odor. To study how different chain transfer agents affect the final product, we performed this process using chain transfer agents such as α-methylstyrene linear dimer, methyl 3-mercaptopropionate and dodecyl mercaptan. A varying dosage of chain transfer agents was added to evaluate concentration effect.

### 2.2. Characterization

Thermogravimetric analysis (TGA, Mettler-Toledo, Zurich, Switzerland) was performed using 5–10 mg ABS resin pellets in the 30–600 °C temperate range at 20 °C /min heating rate under 50 mL/min N_2_ flow rate.

The total VOCs were analyzed according to the standard environmental test method VS-01.00-T-14012-A1-2014. We used thermal desorption–gas chromatography–mass spectrometry (TDS-GC/MS, Agilent, Santa Clara, CA, USA) and a 100 × 100 × 3 mm sample. For this purpose, a 10 L sample bag was filled with 5 L of N_2_, heated to 65 °C and kept at this temperature for 2 h. A Tenax trap was performed at 100 mL/min. A Tenax trap is packed with Tenzx-TA and is flushed with N_2_ for at least 1 h during activation with the flow rate no less than 100 mL/min. During acquisition, the flow rate of the flow pump was 100 mL/min, and the acquisition time was 10 min. After sampling, the trapping tube was taken out and its two ends were sealed. Then, the sample was analyzed as soon as possible by TD-GC/MS.

For odor level testing, a sample was suspended in a glass jar, and an appropriate amount of deionized water was added, after which the jar was sealed, heated to 80 °C and kept at this temperature for 24 h. The odor was evaluated by certified judges. Each sample was given an evaluation grade. It is noted that the odor evaluation is subject to many hardly quantitative factors. Therefore, precautions should be taken for the odor results.

The tensile performance test was carried out according to ISO 527-1:1993 using a universal material testing machine (ZwickRoell GmbH & Co. KG, Ulm, Germany). A sample was prepared on the RR3400 mini vertical injection molding RAY-RAN machine (RAY-RAN, Nuneaton, UK). Tests were performed using 4.0 × 2.1 mm samples at 5.0 mm/min rate and 23 °C. Each resin was tested five times using freshly prepared samples. The value reported in this work represents an average of five measurements.

The Izod impact test was conducted using 695606 cantilever beam impact tester fabricated by CEAST (CEAST, Turin, Italy) using the ISO 180-1993 standard procedure. The experimental conditions were 23 °C, 50 J pendulum and type A spline with a gap 2 mm deep.

Bending strength and flexural modulus tests were performed by a CST4204 microcomputer, controlled by an INSTRON universal testing machine (INSTRON Boston, MA, USA) using the GB/T3356-1999 standard procedure. The sample size was (100 ± 5) × (25 ± 1) × (3.20 ± 0.15) mm. The beam moving speed was 15 mm/min. Three samples were tested per group. The results reported in this work represent an average of these three measurements.

The Rockwell hardness test was conducted by a HR type hardness tester (ZwickRoell GmbH & Co. KG, Ulm Germany) using the ASTM D 785-1998 standard method. The sample size was 80 × 40 × 3.2 mm. Three samples were tested per group. The results reported in this work represent an average of these three measurements.

A heat distortion temperature test was tested by the XRW-300ML instrument (Chengde testing machine Co., Ltd. Chengde, China) using the GB1633-2000 standard procedure. The sample size was 15 × 10 × 4 mm, and the load was 50 ± 1 N.

## 3. Results and Discussions

### 3.1. Effect of Chain Transfer Agents on VOC Content of ABS Resin

To obtain ABS resin with the lowest VOC content, we studied the effect of the type and amount of chain transfer agents on the properties of resulting ABS resin.

In general, a chain transfer agent is the main production ingredient affecting the odor of ABS resin. Currently, most popular chain transfer agents used for the production of bulk and emulsion ABS resins in China and abroad are n-dodecyl- or tert-dodecyl-mercaptans (CAT1). Residual mercaptans emit an easily detectible unpleasant smell. It is especially noticeable during high-temperature 3D printing applications. The odor becomes especially unpleasant because of the release of a large number of small molecules into the surrounding atmosphere and their residual lingering in the resin after its production.

The α-methyl styrene linear dimer monomer (CTA2) is a cationic solid acid catalyst obtained by oligomerization. It can be prepared from a variety of raw materials at a low cost. Its most significant property is odor absence, even if large amounts are used for plastic polymerization. The addition of CTA2 provides a good plasticizing effect and increases the gloss of the final product. Thus, α–methyl-styrene dimer is an ideal linear chain transfer agent for ABS resin fabrication. In fact, it was studied by several research groups for methyl styrene polymerization [12] as well as for styrene-acrylonitrile emulsion copolymerization [1,3]. A continuous drop-wise addition of CTA2 also helped to control the copolymer molecular weight and final composition [13].

Methyl 3-mercapto-propionate (CTA3) has less of an unpleasant smell than n-dodecyl- and tert-dodecyl-mercaptans. Thus, the addition of CTA3 as a chain transfer agent can help adjust the molecular weight distribution and significantly improve the unpleasant resin odor. During polymerization, the mercapto-group forms a three-arm star structure, the presence of which significantly improves chain propagation. Liu et al. [14] pointed out that methyl 3-mercapto-propionate is a very stable monomeric styrene-type chain transfer agent with a long storage shelf-life. Table 1 describes the physical properties of transfer agents tested in this work.

#### 3.1.1. Influence of Chain Transfer Agents on the Thermal Stability of ABS Resin

**Figure 2:**
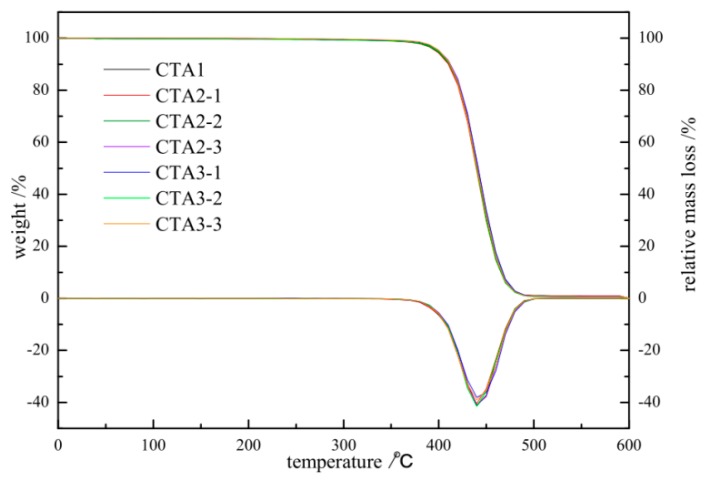
TGA/DTA of ABS resin obtained using different chain transfer agents at different concentrations.

ABS resin obtained using CTA2 and CTA3 chain transfer agents demonstrated better thermal stability than ABS resin obtained with CTA1 (see Figure 2), which is important to prevent resin degradation caused by high operating temperatures as well as oxygen. Higher thermal stability of ABS resins also inhibits the unfavorable formation of acrylonitrile and styrene in the matrix.

Figure 2 shows the integrated thermogravimetric analysis (TGA) and differential thermogravimetry (DTG) curves of ABS prepared under different types and contents of chain transfer agents with N_2_ and a heating rate of 20 °C/min.

The amount of CTA1 is 0.1 mol%. The amount of CAT2-1, CAT2-2, CAT2-3 is 0.3, 0.35, 0.4 mol%, respectively. The amount of CAT3-1, CAT3-2, CAT3-3 is 0.1, 0.25, 0.5 mol%, respectively.

All TGA curves show a sharp weight loss in the 350–500 °C region, which agrees with the previously reported data [15,16]. Thus, the type and amount of chain transfer agent have a good influence on the thermal stability of ABS resin. Changing the type and amount of chain transfer agent has little impact on the thermal stability of ABS resin. Therefore, CTA2 and CTA3 can be selected instead of CTA1 for ABS polymerization experiments.

#### 3.1.2. Influence of Different Chain Transfer Agents on Mechanical Properties and Odor Levels of ABS Resins

**Figure 3:**
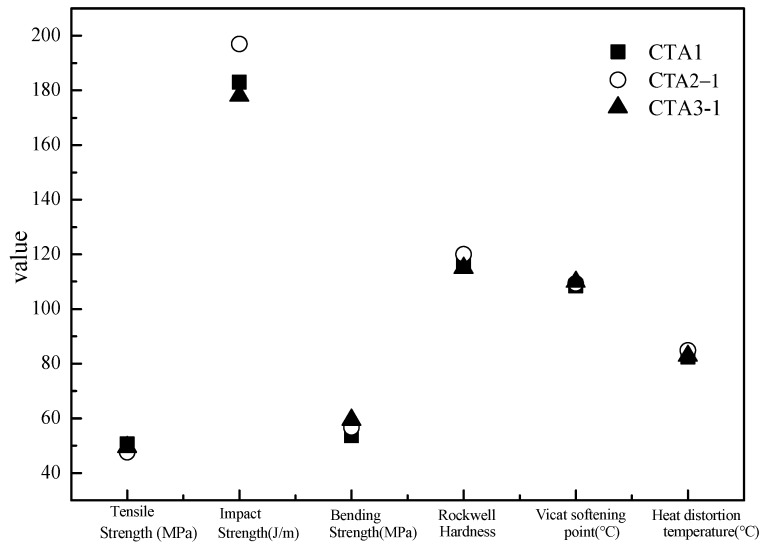
Effect of different chain transfer agents on properties of the resulting ABS resins.

**Figure 4:**
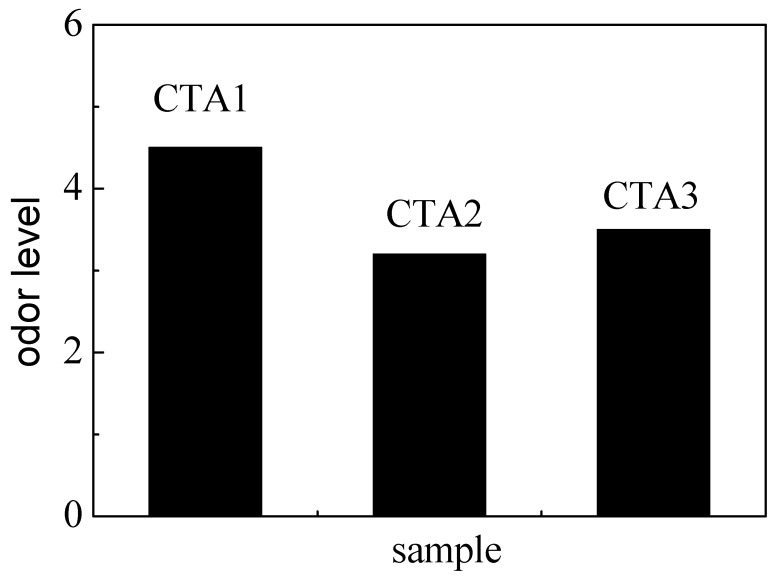
Effect of different chain transfer agents on the odor level of the resulting ABS resins.

Mechanical properties of ABS resin samples obtained using CTA1, CTA2 and CTA3 chain transfer agents were very similar to each other (Figure 3). However, the chain transfer agent choice affected ABS resin smell. According to the DPCA odor intensity scale, ABS products obtained using CTA2 and CTA3 agents demonstrated a pleasant smell (Figure 4). Furthermore, ABS resin containing CTA2 smelled slightly better than ABS resin prepared using CTA3. ABS resin obtained with the CTA1 agent had the worst odor mostly because CTA1 contains thiol functional groups.

Thus, usage of non-thiol chain transfer agents, such as α-methyl styrene linear dimers, can help to eliminate the unpleasant odor of the ABS resin and control the final product performance matrix.

#### 3.1.3. The Effect of Chain Transfer Agent Content

**Figure 5:**
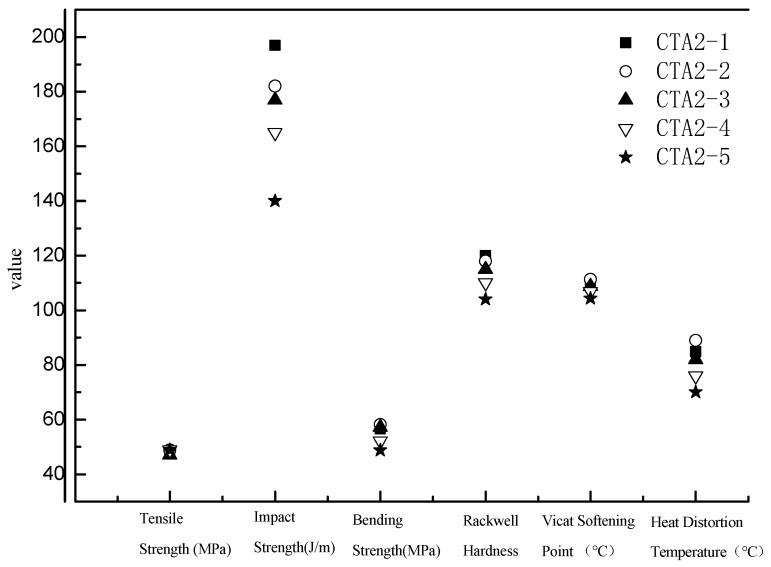
Influence of α-methyl styrene content on ABS resin performance.

**Figure 6:**
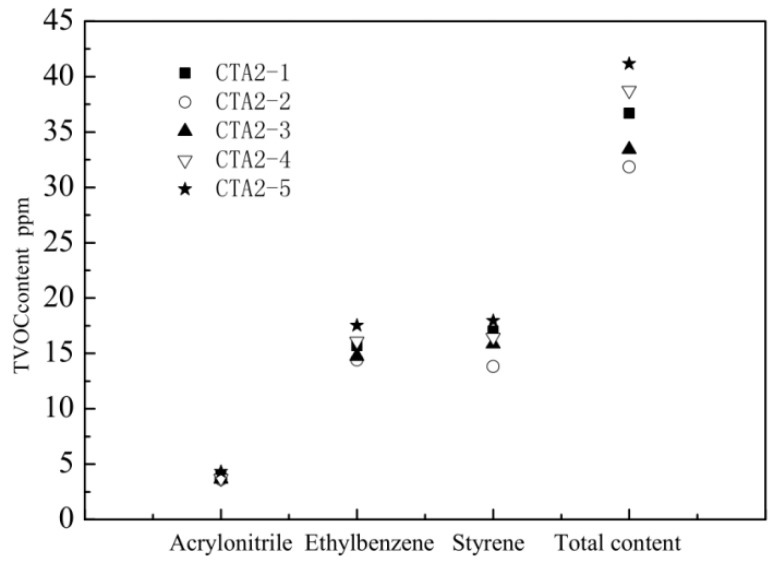
Influence of α-methyl styrene content on the amount of different VOC and on TVOC.

ABS resins prepared using methyl-styrene dimer as a chain transfer agent showed the lowest total VOC (TVOC) content. Therefore, we further studied how the methyl-styrene dimer amount affected mechanical properties and the TVOC content of ABS resin. These properties were also compared with the properties of ABS products obtained using the standard dodecyl mercaptan chain transfer agent (Figure 5 and Figure 6).

The amount of CAT2-4 and CAT2-5 is 0.45 and 0.5 mol%, respectively.

When the content of α-methyl styrene linear dimer was three times the original dodecyl mercaptan chain transfer agent content (sample CTA1 0.1 mol% in inlet amount), the ABS resin showed the best mechanical properties and the lowest TVOC content. Thus, α-methyl-styrene dimer is an ideal chain transfer agent for ABS, which effectively regulates the molecular weight of the continuous phase and improves the viscosity of the prepolymer. It provides ABS resin with impact properties significantly better than thiol-based agents do. ABS resin with narrow molecular weight distribution possesses excellent fluidity and mechanical properties.

### 3.2. Supercritical Extraction of VOC from ABS Resins

Supercritical extraction technology was adopted during the mixing stage of the extrusion process. The supercritical fluid was injected into a twin-screw extruder and mixed with molten ABS resin. Since CO_2_ is chemically stable and inexpensive, it was selected as the extractant. The critical temperature and pressure of extractant CO_2_ are 31.06 °C and 7.39 MPa.

The preparation of low-VOC ABS resin by supercritical extraction was optimized using the orthogonal method. We measured the TVOC content and odor reduction after the supercritical extraction of ABS resins. The primary influencing parameters included extrusion temperature (A), residence time (B), vacuum degree (C) and CO_2_ dosage (D). The amount of extractant was expressed as a percentage of the total amount of ABS resin produced. Each parameter had three evaluation levels (see Table 2). The final TVOC content in the ABS resin was used as an evaluation index (see Table 3).

Table 4 and Table 5 show that extrusion temperature, residence time, vacuum and amount of extractant (parameters A–D, respectively) significantly affect TVOC content in ABS resin (P < 0.01). Their influence can be expressed in the following order: D > C > A > B. The best extraction process was at A–D values equal to A_3_, B_1_, C_3_ and D_2_, i.e., 250 °C extrusion temperature, one min residence time, −0.099 MPa vacuum, and 1.5% CO_2_ content. The resulting TVOC content of the ABS product processed at these optimized conditions was ~25 ppm.

**Figure 7:**
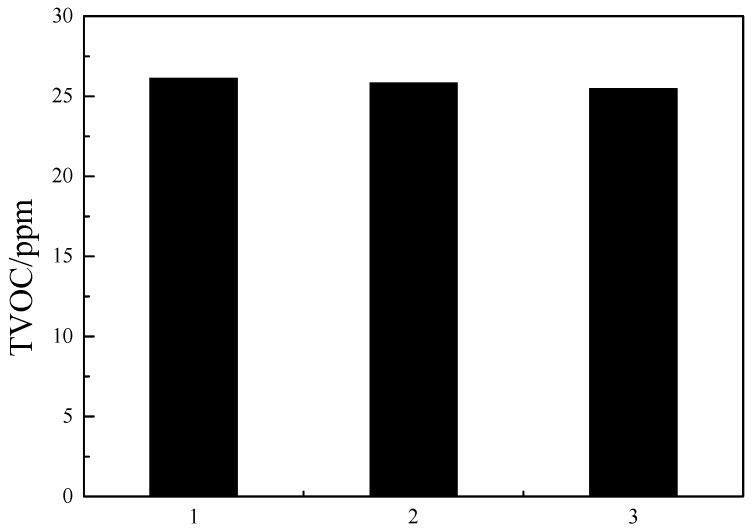
Results of confirmatory experiment.

The best parameters obtained from the orthogonal test were validated experimentally using commercial ABS reins (see Figure 7). The TVOC content in these ABS resins was below 50 ppm, which confirms the reliability of our orthogonal tests.

## 4. Conclusions

In order to reduce residual VOC and improve the disagreeable odor characteristics of ABS resin products, this paper presented a novel synthesis method. An innovative chain transfer agent coupled with a unique supercritical extraction process has been evaluated. Interestingly, it also shows promise in large scale production of low VOCs/odor ABS resin. With the comparable thermal stability, ABS resins prepared using chain transfer agents of α-methyl-styrene linear dimer and methyl 3-mercapto-propionate showed significantly lower residual VOC/odor as compared to what dodecyl mercaptan did. In particular, when α-methylstyrene linear dimer content was 0.3% (mol), the prepared ABS resin demonstrated the best overall characteristics. An orthogonal method was implemented as well to optimize ABS resin preparation conditions. The best operating conditions in the supercritical extraction process were 250 °C extrusion temperature, one minute residence time, minus 99 KPa vacuum and 1.5% of CO_2_ extracting agent.

## Figures and Tables

**Table 1:** Physical properties of transfer agents used in this work.

Name	CTA2	CTA3	CTA1
Molecular formula	C_9_H_10_	C_4_H_8_O_2_S	C_12_H_26_S
Molecular weight	118.18	120.17	202.40
Exterior	Light yellow viscous liquid	Colorless-very light yellow transparent liquid	Colorless or light yellow liquid

**Table 2:** Preparation of ABS resin with low VOC using supercritical fluid extraction.

Level	Parameters			
	A	B	C	D
	Temperature, °C	Residence Time, min	Vacuum Degree (C), MPa	CO_2_ Content, %
1	240	1	−0.095	1
2	245	1.2	−0.097	1.5
3	250	1.5	−0.099	1.8

**Table 3:** Orthogonal experimental design and results.

Test Number	Setup Conditions				Result
	Temperature	Residence Time	Vacuum Degree	CO_2_ Content	TVOC Content (ppm)
1	240	1	−0.095	1	49.325
2	240	1.2	−0.097	1.5	36.697
3	240	1.5	−0.099	1.8	47.641
4	245	1	−0.097	1.8	44.714
5	245	1.2	−0.099	1	29.092
6	245	1.5	−0.095	1.5	38.168
7	250	1	−0.099	1.5	25.317
8	250	1.2	−0.095	1.8	59.356
9	250	1.5	−0.097	1	52.958

**Table 4:** Process orthogonal test analysis results.

Parameters					
	A	B	C	D	
Parameter	Temperature	Residence Time	Vacuum Degree	Extractant	Experimental Result
1	1	1	1	1	49.325
2	1	2	2	2	36.697
3	1	3	3	3	47.641
4	2	1	2	3	44.714
5	2	2	3	1	29.092
6	2	3	1	2	38.168
7	3	1	3	2	25.317
8	3	2	1	3	59.356
9	3	3	2	1	52.958
Mean 1	44.554	39.785	48.950	43.792	
Mean 2	37.325	41.715	44.790	33.394	
Mean 3	45.877	46.256	34.017	50.570	
Range	8.552	6.471	14.933	17.176	

**Table 5:** Analysis of variance of orthogonal test results.

Parameter	Sum of Squared Deviation	Degree of Freedom	Variance	F	P
Temperature A	127.160	2	63.580	1.921	<0.01
Residence time B	66.206	2	33.103	0.999	<0.01
Vacuum C	356.358	2	178.179	5.382	<0.01
Extractant D	449.088	2	224.544	6.783	<0.01
error	66.21	2	33.105		

Note: F _0.01 (2,2)_ = 9.00.
